# The multifactorial aquaculture‐related COVID‐19 shock in Benin, West Africa: A socio‐economic perspective of mitigating the disruptive impacts on the small‐scale and subsistence producers

**DOI:** 10.1002/aff2.78

**Published:** 2022-10-24

**Authors:** Toundji Olivier Amoussou, Comlan Eugène Dessouassi, Dorothé Ngondjeb Yong, Siméon Mahougnon Fagnon, Luc Houngbe, Vinsoun Millogo, Ibrahim Imorou Toko, Emmanuel A. Frimpong

**Affiliations:** ^1^ Higher Institute of Sustainable Development University of Fada N'Gourma Fada N'Gourma Burkina Faso; ^2^ National Aquaculture Development Programme, Ouémé‐Atlantique‐Littoral‐Mono Territorial Agricultural Development Agency Ministry of Agriculture, Livestock and Fisheries Cotonou Benin; ^3^ Faculty of Economics and Management University of Yaoundé II Yaoundé Cameroon; ^4^ Phytosynthese Mozac France; ^5^ Agriculture Innovation Lab, Appropriate Scale Mechanization Consortium, Institute of Rural Development Nazi Boni University Bobo‐Dioulasso Burkina Faso; ^6^ Research Unit in Aquaculture and Aquatic Ecotoxicology, Faculty of Agronomy University of Parakou Parakou Benin; ^7^ Department of Fish and Wildlife Conservation Virginia Polytechnic Institute and State University Blacksburg Virginia USA

**Keywords:** aquaculture value chain, aqua‐farmers, assessment, inputs, sustainability

## Abstract

Aquaculture development in Benin depends heavily on small‐scale and subsistence aquaculture producers (SSAPs), who are sustaining production activities. The aquaculture sector is vulnerable to market shocks because of its dependence on inputs and equipment, which are mainly imported from overseas. The recent outbreaks of global aquaculture diseases have also proven the sensitivity of the sector. The vulnerability of SSAPs is expected to increase as a subsequent result of the coronavirus disease 2019 (COVID‐19) pandemic. This is the reason why COVID‐19‐related difficulties affecting aquaculture production deserve to be evaluated in the country's aquaculture sector. We conducted an online survey to assess the impact of COVID‐19 and the resulting restrictive measures on the functioning of SSAPs farms. Data were collected from 98 SSAPs informants spread over the geographic area with high aquaculture production potential in the country. The rate of increase in input prices, linear discriminant and factorial correspondence analyses projected severe constraints in almost all the value chain. The rates of increase in the unit price of inputs increased from 14% to 188%, thus weakening aqua‐farmers’ purchasing capacity. COVID‐19 has led to a drop in the sales turnover of aqua‐farms, resulting in staff reductions and unemployment. Difficulties in accessing quality inputs have led to the disruption of fish growth and thus the production cycle. The sale of aquaculture products saw a 20%–30% drop in turnover in many farms. The critical challenges mentioned by both men and women SSAPs are mainly the high cost of fish feed, the rising input and transport costs, and the lack of financial resources. Therefore, short‐, medium‐, and long‐term mitigation measures are suggested and could help to alleviate these difficulties while sustaining the blue revolution already under way. This community of men and women SSAPs should adopt and strengthen their use of existing endogenous technologies as well as digital modern options for remote aquaculture sales, in order to cope with future disruptions. The scientific community should help to propose incentive‐based alternative options (e.g., affordable production systems, and efficient micro‐credit model) that can motivate SSAPs to continue with aquaculture, as a guarantee of the sustainability of their livelihoods.

## Graphical Abstract

This study deals with a timely topic, COVID‐19, whose impacts on aquaculture have never been quantified in Benin. Direct discussions were held with aquaculture producers to understand how they are feeling the impact of the pandemic on their business. The study focused on small‐scale aquaculture producers who are the ones helping to spread the Blue Revolution throughout the country. The proposed solutions are innovative and will help build the resilience of the various stakeholders in the face of COVID‐19 in Benin.

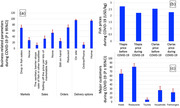

## 1 INTRODUCTION

Since its declaration in March 2020, the coronavirus disease 2019 (COVID‐19) pandemic has caused huge loss of human life of more than 5.4 million, as of 26 December 2021 (WHO, 2021) and economic disruption across all spheres of development (CRS, 2021). The context of the COVID‐19 crisis accentuates the situation of food insecurity and malnutrition (Love et al., 2021) of communities of many countries. Governments around the world face multiple challenges related to reducing the devastating health impact and protecting lives, livelihoods, and ensuring an adequate food supply and functioning services for those most in need (FAO, 2020a; Siche, 2020). Commercial fisheries and aquaculture sectors globally experienced numerous and significant perturbations during the early months of the COVID‐19 pandemic, affecting the livelihoods of millions of actors worldwide (Mangano et al., 2022; Smith et al., 2020). In some countries in Asia and Africa, the aquatic food value chains were temporarily severely disrupted, but impacts on demand for aquatic foods, production inputs, and labour have been longer lasting than impacts on their supply (Belton et al., 2021). In the Maghreb, fishery and aquaculture sectors experienced a significant decline in activity in 2020 (FAO, 2020c; Jlassi et al., 2021). In the wake of the COVID‐19 crisis, other climatic calamities have affected ecosystems and livelihoods, especially those of fishers who depend on fishing as their sole source of income in Kenya (Aura et al., 2020) and also on a global scale (Sarà et al., 2022). In Ghana, the crisis has reduced incomes for most actors along the aquaculture value chain and is anticipated to reduce future production as there are disruptions in input and output markets (Ragasa et al., 2021).

The advent of the COVID‐19 pandemic has led the Beninese government to adopt restrictive measures that were, among others: (i) establishment of a sanitary barrier zone (SBZ) restricting movement between the most exposed localities, (ii) banning of the circulation of buses and minibuses for public transport, (iii) entry restrictions on land and air borders, (iv) closure of churches, mosques and other places of worship, bars, discos, beaches, and other places of social activity (Benin Government, 2020). While the measures put in place have been necessary to reduce the spread of the virus and the immediate loss of human life, they have had negative impacts on economic activities such as aquaculture. Lockdowns and COVID‐19 restrictions have been shown to have significant impacts on other domains of human activity, including food and nutrition security, jobs, livelihoods, gender equality, and potential social unrest (Ejeromedoghene et al., 2020; Kansiime et al., 2021). Of course, it is important to acknowledge the importance of the restrictions for preventing loss of life, as the risk to workers in aquaculture, fisheries, and seafood processing sectors around the world would have been worse without some kind of restrictions (Belton et al., 2021). However, lockdowns and restrictions have taken their toll on all sorts of economic activity, including seafood systems (Belton et al., 2021).

This pandemic and all forms of strategies for its management have been disruptive to the production and supply system of small‐scale and subsistence aquaculture producers (SSAPs). In Benin, the aquaculture sector contributes 11.31% to agricultural Gross Domestic Product (GDP) and 3% to national GDP (Rurangwa et al., 2014). With a non‐existent mariculture, the inland aquaculture production increased from 308 tonnes in 2009 to 5115 tonnes in 2018 (Amoussou et al., 2020). Such a domestic production fails to meet the needs of the population. And the country remains 56.09% dependent on imports for certain fisheries products (MAEP, 2020). The situation of COVID‐19 constitutes a real threat to food security in its current state, as the national fisheries and aquaculture production can only cover an average of 4.47 kg/capita/year. For example, in 2007, the annual consumption of fish was estimated at 12 kg/capita/year (FAO, 2008) for a minimum recommended quantity of 30 kg/capita/year, that is, a coverage rate of 40% of people's protein needs from fish. The shortfall in fish protein is being made up by ever‐increasing imports amounting to 169,363.96 tonnes in 2016 (MAEP, 2019). All over the country, the SSAPs employ over 59,188 people (DPH, 2019) and 93% of aqua‐farms are managed by men (Amoussou et al., 2017). In contrast to 93% of aquacultures being managed by men, women are involved in crab and oyster fishing activities (FAO, 2008). In addition, approximately 40,000 women are active in the wholesale trade, processing, and marketing of fishery products (Rurangwa et al., 2014). The processing of fishery products (smoking, salting, fish fillets, etc.) is mainly carried out by women fish workers (WFW), who most often walk along all the main roads of the big cities to sell products to consumers.

In the model of the aquaculture value chain in Benin, the SSAPs usually purchase inputs (e.g., fish feed, seeds, vaccines, antibiotics, pond supplements) from large‐scale aquaculture producers. SSAPs are driving development of aquaculture, as the aquaculture development model of the country focuses on the roll‐out of pilot SSAPs that serve to mentor the other SSAPs (DSID, 2019). With the COVID‐19‐related restrictive measures, the large‐scale producers have restricted their sales of inputs to the SSAPs. This situation makes the SSAPs more vulnerable to the COVID‐19‐related market shocks. Yet, the SSAPs are the ones who are helping to catalyze aquaculture production in the country. In addition to that, changes in the contribution of the SSAPs to GDP could be expected. Therefore, the situation urgently calls for collective action by governments, development organizations, non‐governmental organization (NGOs), donors, the private sector, and researchers to mobilize rapid support (Bennett et al., 2020) in addressing COVID‐19 business challenges for the SSAPs. We are targeting SSAPs as a new mechanism for empowering their communities. Clearly, we need to separate the effects due to COVID‐19 from those due to other sources (e.g., starvation). This means that such actions can only have a real impact if decisions are based on concrete data from these SSAPs.

With the aim to help the SSAPs and decision‐makers cope during the pandemic, this study was carried out on the basis of a prospective analysis of the impact of COVID‐19 on the performance and future economic vitality of SSAPs. For this purpose, we first present the study area and methodologies. Second, we summarize information obtained from both quantitative and quantitative analyses across the entire aquaculture value chain. Third, we propose mitigation strategies in order to limit the risks SSAPs are facing. We conclude with short‐, medium‐, and long‐term mitigation recommendations to support a suitable and equitable strategy to combat the pandemic, as the aquaculture value chain plays a central role in supporting livelihoods and nutrition security.

## 2 METHODOLOGY

### 2.1 Study area

**FIGURE 1:**
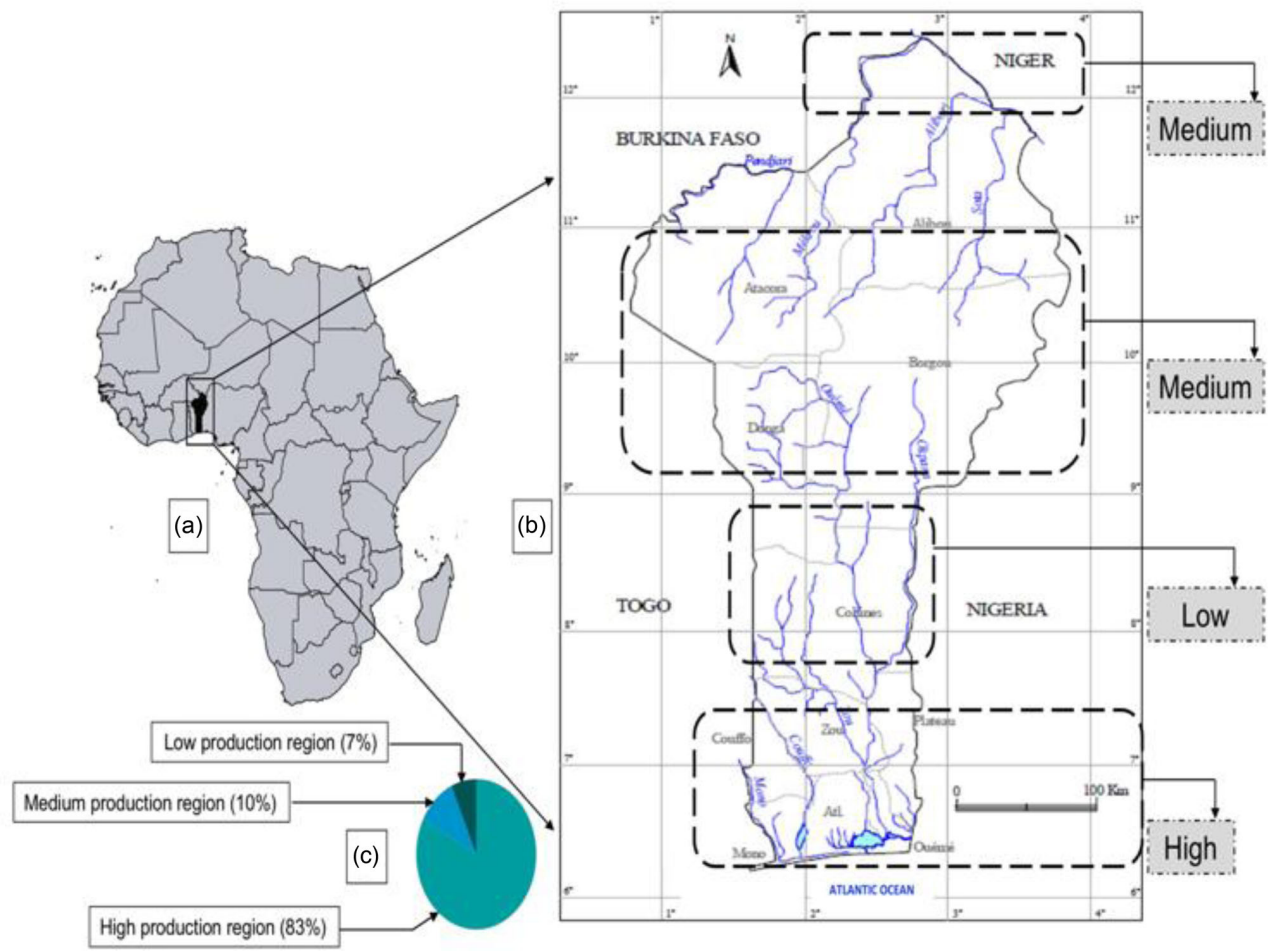
Map of the hydrographic network of Benin, along with the regional breakdown of the potential for aquaculture development: (a and b) the African regions and Benin country, respectively. Dotted demarcations highlight areas of high, medium and low aquaculture production. The accompanying pie‐chart (c) is the combined proportion of both small‐ and large‐scale aquaculture producers in the three regions.

This study was conducted in Benin, a country with a land area of 114,763 km^2^, located in the Gulf of Guinea in West Africa, between 9°30′N, 2°15′E. The country is subdivided into three zones of varied fisheries and aquaculture potentiality with a high potential in the South, a low potential in the Centre, and a medium potential in the North. The low production area is characterized by the presence of many hills and mountains. Of a total of 1788 small‐ and large‐scale aquaculture producers in the country in 2020, 1481 (83%) are active in the southern part of the country, 185 (10%) in the central part, and 122 (7%) in the northern part (DPH, 2021). The country has a fairly large hydrographic network covering an area of 130,000 ha (Figure 1), and the water bodies of Southern Benin are teeming with 85% of fishers, compared to 14% in the Centre and 1% in Northern Benin (DPH, 2019).

The SSAPs in Benin mostly use aquaculture production systems consisting of traditional equipment such as ponds, concrete tanks. Modern equipment such as recirculating aquaculture systems, plastic tanks, fixed cages, and floating cages are used by large‐scale aquaculture producers. The volumes and areas of these facilities vary from one SSAP to another (Table 1). The most commonly cultured fish species belong mainly to families Cichlidae and Clariidae. The local feed producers use small mills, grinders, mixers, and shovels/grinders operating on generator power. The feeds are sun‐dried after pelleting. As far as the processing of fishery products is concerned, many processing units (processing ovens, FAO‐Thiaroye ovens) are being set up across the country (Table 1).

**TABLE 1:** Characteristics of aquaculture production and processing systems, as well as aqua‐feed manufacturing tools in Benin

Item	Description
Traditional aquaculture activity	Pond, concrete tank, Whedo, Acadja, enclosure, agro‐aquaculture
Modern aquaculture activity	Recirculating aquaculture system, floating cage, plastic tank, aquaponics
Fish species used in aquaculture	Cichlidae: The Nile tilapia Oreochromis niloticus (Linnaeus, 1758), Mozambique tilapia Oreochromis mossambicus (Peter, 1852), Blackchin tilapia Sarotherodon melanotheron melanotheron Rüppell, 1852, Guinean tilapia Coptodon guineensis (Günther, 1862)
	Clariidae: North African catfish Clarias gariepinus (Burchell, 1822), Sampa Heterobranchus longifilis Valenciennes, 1840
Feed mills	Feed producers use small mills, grinders, mixers, and shovels/grinders operating on generator power
	The feed is sun‐dried
Processing units for fishery products	No specific categories, they are generally fishmongers who work/process the products
	The situation is evolving with a recent installation of processing ovens in the locality of Ifangni
	A fish smoking unit based on the FAO‐Thiaroye processing technique, consisting of Thiaroye Processing Ovens has been set up in the village of Gbaglan‐Ganfan in the commune of Avrankou
Facility area or volume	Cylindrical above ground plastic tank (0.01–10.60 m^3^)
	Non‐cylindrical above ground plastic tank (0.75–240 m^3^)
	Concrete tank (0.8–1200 m^3^)
	Fixed cage (3–600 m^3^)
	Floating cage (9–4500 m^3^)
	Enclosure (116–1000 m^2^)
	Drainable pond (12–8000 m^2^)
	Non‐drainable pond (15–29700 m^2^)

*Source*: Fisheries Department of the Ministry of Agriculture, Livestock and Fisheries of Benin.

### 2.2 Aquaculture enterprise and farm surveys

Given the COVID‐19 health situation, the geographical distribution of SSAPs and the fact that we did not have telephone contact for all the SSAPs, we opted for a cluster sampling. SSAPs are those predominantly engaged in traditional aquaculture with very modern inputs (e.g., commercial fish feed, improved seeds, vaccines, antibiotics, pond supplements). There are no clear statistics on the functional SSAPs regularly active in the country as the above‐mentioned statistics include both small‐ and large‐scale aquaculture producers. The study was limited to those who agreed to participate in the survey. We noted the reluctance of many SSAPs to complete the online survey, probably due to their digital illiteracy. They were therefore contacted individually to have the form completed for them on the basis of a phone call, a printed version of the questionnaire. Difficulties with cell phone coverage also made it difficult to talk to some SSAPs. The study was carried out on a set of 98 of the SSAP market ecosystems of Benin. The sample size of the survey was determined assuming 0.54% of the total population of the country declared themselves SSAPs, while using the Schwartz and Denne's (2006) approach:

(1) $$N=\left(eZ^{2}pq\right)\;/i^{2},$$

where *N* is the sample size, *e* is the anticipation of non‐response, *i* is the precision at 5%, *Z* is the standard deviation for 5% error, and *p* is the incidence of population of SSAPs (0.54% of total population) in 2020; *q* = 1 – *p*.

The research methodological approach was applied following that of Murray et al. (2016). A questionnaire was drawn up on the basis of a systematic literature review carried out focusing on keyword searches. The keyword searches were based on Boolean commands that have been established by combining the following letters: “coronavirus, COVID‐19, pandemic, impact, aquaculture, aquatic, seafood, food, feed, fish, fishing, fishery, fisheries, gender, man, woman, women, youth, shrimp, vulnerability, resilience, adaptation, farm, farming, business, consumption” and by combining them using Boolean terms (i.e., AND, OR). The questionnaire was then adjusted by some technical agents of the Fisheries Department of the Ministry of Agriculture, Livestock and Fisheries in order to better reflect the real difficulties of the SSAPs. The field research was thus undertaken through survey questions to determine how SSAPs are coping with this COVID‐19 pandemic. Since the country was in lockdown, field data were collected through phone interviews, social media (WhatsApp, Facebook Messenger) interviews, or many other means (on paper and email), considering physical distance and travel restrictions as done in previous studies (e.g., Arthur et al., 2018; Dehnen‐Schmutz et al., 2016; Geldsetzer, 2020). Data collection through digital tools (e.g., online instruments, smartphone, online datasets, questionnaire) is known to be effective as evidenced by numerous studies conducted recently on the impact of COVID‐19 (e.g., Azra et al., 2021; Lebel et al., 2021; Love et al., 2021; Sarà et al., 2022; Smith et al., 2020; Stoll et al., 2021; White et al., 2021). Therefore, use of digital tools does not affect the accuracy of the responses provided by our SSAPs.

**FIGURE 2:**
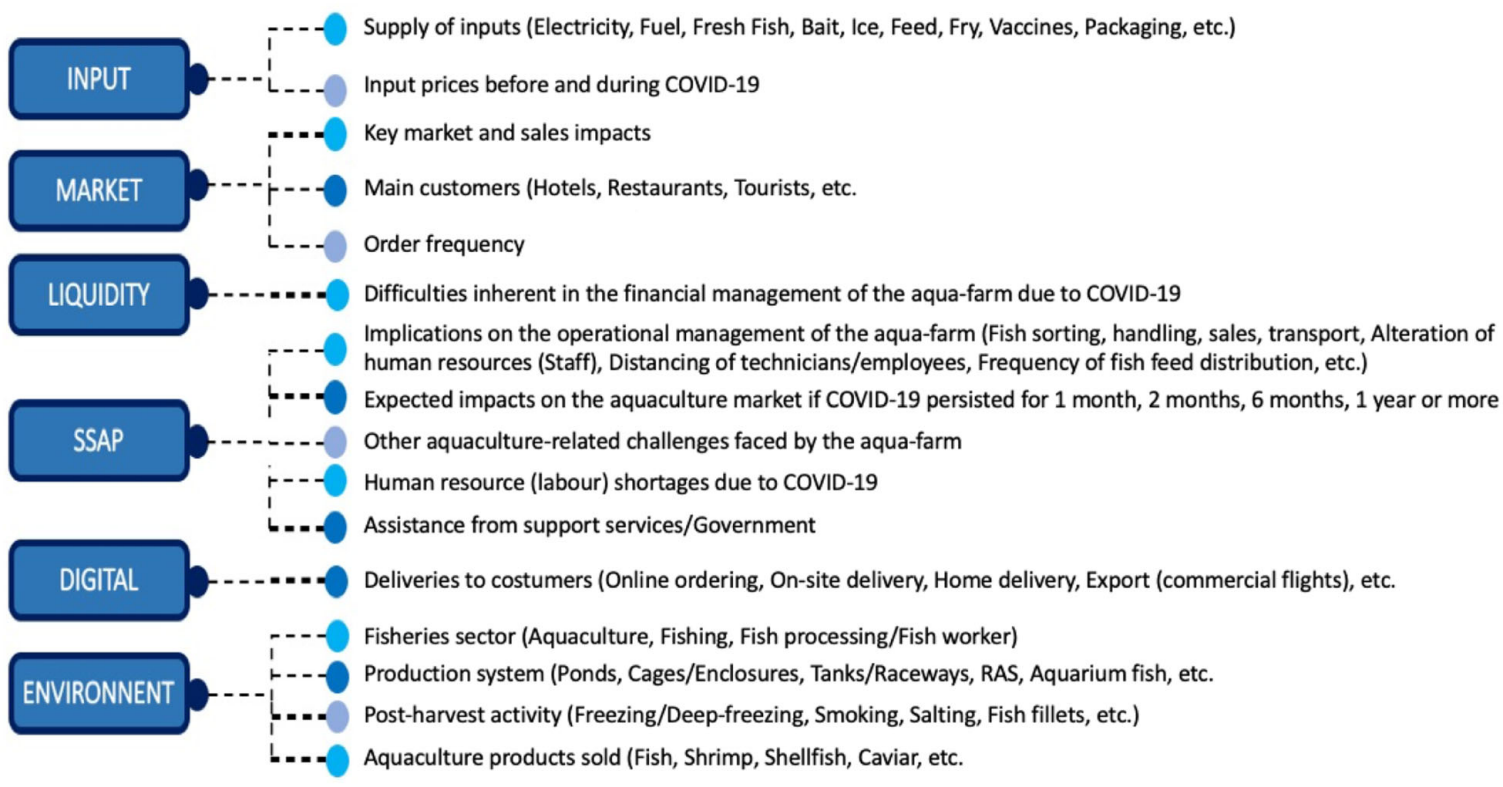
Summary of key COVID‐19‐related questions administered to stakeholders. Different colours have been used to differentiate between the key points addressed in the survey form under each of the headings of the aquaculture value chain. Abbreviations: COVID‐19, coronavirus disease 2019; SSAP, small‐scale and subsistence aquaculture producers

As suggested by Bennett et al. (2020), examples from published media, policy organizations, and public sources were also used to provide insights into the impacts that the SSAP sector is experiencing all over the country. The knock‐on effects from restrictive measures were analyzed based on input supplies, sales, customers, orders, deliveries, financial management, operational management, human resources, and supports received from government. The main COVID‐19‐related drivers considered at each aquaculture enterprise and farm (AEF) are summarized in Figure 2. The ethical consideration of this study is based on the fact that all SSAPs had been informed of the purpose of the study and that their participation was voluntary and anonymous. Verbal consent was obtained from each SSAP and documented in the questionnaire. The survey form consisted of both pre‐coded and/or open‐ended questions.

We also investigated whether COVID‐19 could have an impact on aquaculture and its market at the national level if it persisted for 1 month, 2 months, 6 months, 1 year, so on. Information on aquaculture sector, type of activity, fishery products sold, input prices before and during COVID‐19, main customers, delivery modes to customers, and future impact of COVID‐19 were pre‐coded. For the type of aquaculture activity, the SSAPs were given the choice to tick off aquaculture, or processing (Table S1). The type of activity concerned aquaculture in ponds, cages/enclosures, tanks/raceways, so on. For the processing activity, the boxes to be selected were: deep‐freezing/freezing, smoking, salting, fish fillets, so on. The fishery products sold included fish, shrimps, shellfish, caviar, so on. The inputs initially listed were electrical energy, fuel, fresh fish, fish bait, ice, fish feed, fry, vaccines, antibiotics, packaging, so on. Each SSAP therefore listed the impacted inputs and gave their purchase price before and during COVID‐19. The main customers to be selected were hotels, restaurants, tourists, households, fishmongers, so on. The modes of delivery offered to the informants were (i) on‐site delivery, (ii) on‐site delivery after an online order, (iii) home delivery, (iv) export (commercial flights), so on. We considered the time‐frame deadline between the pre‐COVID‐19 period and the during‐COVID‐19 period to be March 30, 2020, when the SBZ was mandated.

The open‐ended questions focused on (i) the impact of COVID‐19 on input supplies, (ii) the main impacts related to sales, (iii) the effectiveness of orders, (iv) the difficulties inherent in the financial management of the farm/activity due to COVID‐19, (v) the implications on the operational management of the farm/activity, (vi) the aquaculture‐related diseases that the farm is facing, (vii) the human resource shortages due to COVID‐19, and (viii) advice from extension services. We asked each SSAP to specify the implications of COVID‐19 on the operational management of the farm/activity (e.g., length classification, fish removal, juvenile handling, sales, transport, alternation of human resources, distancing of employees, frequency of feeding).

### 2.3 Data analysis

The rate of increase approach was employed to find out the critical constraints faced by the aquaculture hatcheries and processors. The rate of increase in the purchase price of aquaculture inputs was calculated using the formula:

(2) RateofIncrease=(“Finalvalue−Initialvalue”/“Initialvalue”)×100.

The confidence level of each proportion (*P*) has been estimated by a 95% confidence interval (CI) so that the results are presented in tables and figures as *P* ± CI%. The 95% CI was computed by the formula:

(3) $$\mathrm{CI}=1.96\surd \left(\left(\left[P\left(1-P\right)\right]N\right)\right),$$

where *P* is the proportion and *N* is the sample size.

In order to have an overview of the perception and the different positions taken by SSAPs informants on the impacts of COVID‐19, a linear discriminant analysis (LDA) of the latent variables and a factorial correspondence analysis (FCA) was carried out using R Studio Version 1.3.1093 (RStudio Team, 2020). The choice of LDA is explained by the fact that all the variables selected for the analysis of sales perceptions were qualitative. We used LDA to reduce the dimensionality of the dataset to understand the influence of different variables to the separability of data across territorial departments. The aim was therefore to find the hidden variables that best describe our data. The equation of the mathematical model used was fitted as:

(4) $$y_{\mathrm{ijkl}}=\alpha_{i}+\beta_{j}+\lambda_{k}+\delta_{l}+\xi_{\mathrm{ijkl}},$$

where *y*_ijkl_ is a SSAP's perception in the *i*th department, on the *j*th sale option, the *k*th order option, and the *l*th delivery option. ξ_ijkl_ is the random error associated with the *y*_ijkl_ exploratory model investigated.

The territorial department was considered as response or explanatory variable (i.e., positively known classes to be modelled), while the other variables were considered as predictor or latent variables. Here, the predictors considered were the market‐related impact, the sale‐related impact, the tilapia price before COVID‐19, the tilapia price during COVID‐19, the catfish price before COVID‐19, the catfish price during COVID‐19, the major customers, the orders still on hand, and the delivery options. The categories assigned to the response variable are Atlantique, Ouémé, and Mono. So, LDA was carried out in order to predict how the SSAPs in each department of the country felt about the sales of aquaculture production, if COVID‐19 continued. The lda function of the package MASS made it possible to realize the LDA. Since no a priori is available, the probabilities were estimated by the proportion of observations in each territorial department. Interpretations were thus made by analyzing the influence of the coefficients of linear discriminants on the score. The scores considered here are those above 2. We further conducted a factor analysis to understand (i) the correspondence of how COVID‐19 could seriously impact the aquaculture/fish market if it still persisted at 1 month, 2 months, 6 months, 1 year, and (ii) the correspondence of the different advices received from NGOs and government departments, across localities during the pandemic. The FCA was considered because the variables chosen to study the types of support or advice received by the SSAPs were all qualitative. So, the FCA was carried out in order to cross‐check the support or advice received by the SSAPs during COVID‐19 between the different localities. This analysis considered the real moment when the impact of COVID‐19 would be felt in the SSAPs, the advice or support provider and the type of advice provided. To perform the FCA, the ca function of the FactoMineR package was used.

## 3 RESULTS AND DISCUSSION

The results are presented and discussed in six sections on the overall impact of COVID‐19 and the economic loss to the aquaculture industry as a whole. Mitigation measures have been suggested in order to lessen the observed impacts.

### 3.1 Access to inputs

**FIGURE 3:**
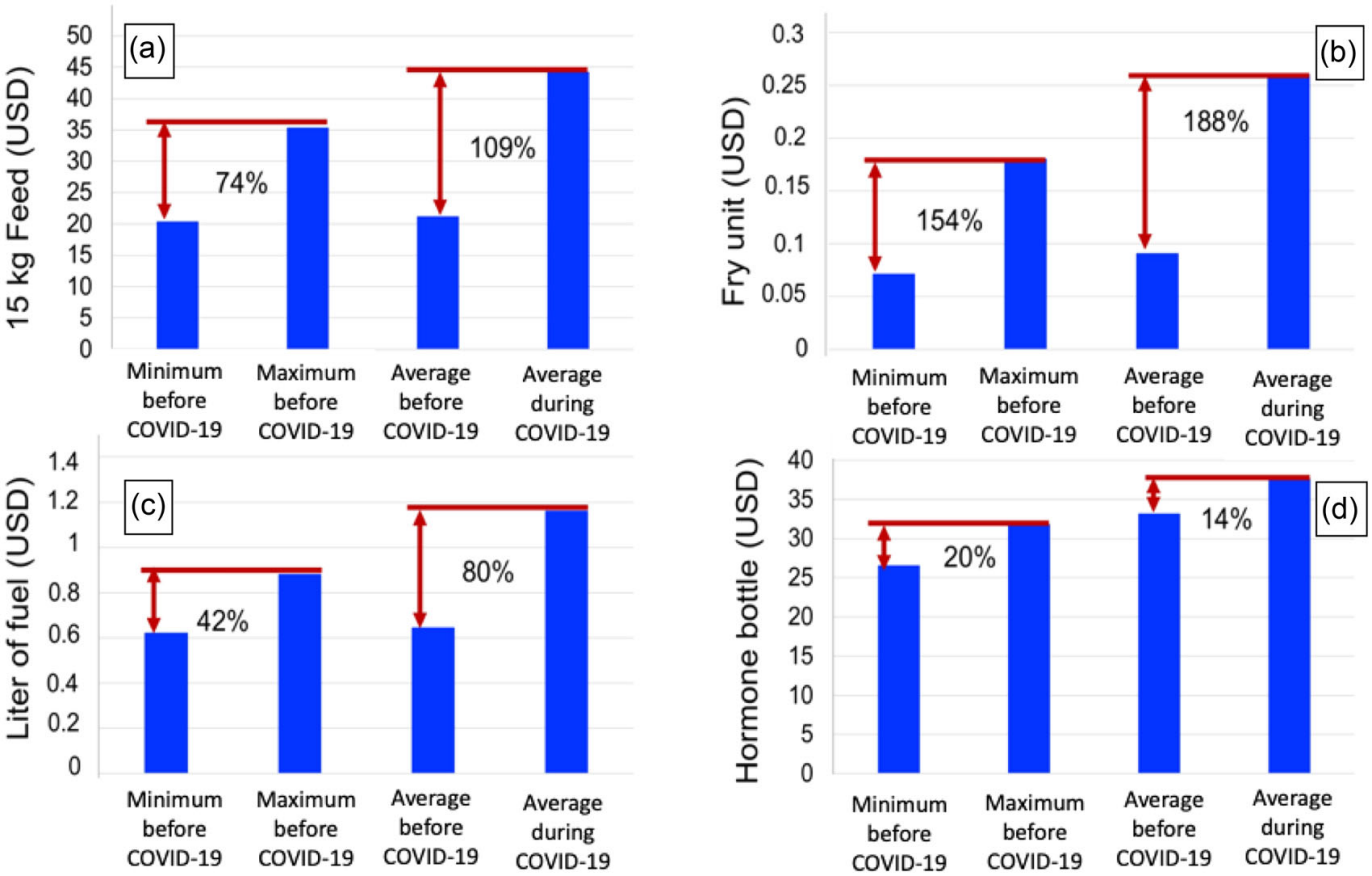
Acquisition prices (USD) of aquaculture inputs before and during COVID‐19 along with their rates of increase. This analysis was done on the basis of the rates of increase in the buying prices of the four inputs. Abbreviations: COVID‐19, coronavirus disease 2019; USD, United States dollar

The aquaculture inputs that have been impacted by the advent of COVID‐19 are fish feed, seed, fuel, and hormone OVAPRIM^®^ (Syndel, Canada). The rates of increase in the unit price of commercial 15 kg feed, unit of seed, and litre of fuel increased from 74% to 109%, 154% to 188%, and 42% to 80%, respectively. As for the hormone bottle, the rate of increase decreased from 20% to 14%. Nevertheless, it is important to note that the purchase price of all these inputs has increased during COVID‐19 (Figure 3). Difficulties of access to inputs and stock shortages were felt by SSAPs, throughout the country. The primary reported constraints were those related to the imports and supplies of inputs (especially fish feed, fuel, and hormones), due to the closure of land borders by the countries of the West African sub‐region. Indeed, these constraints are linked to difficulties in supplying importers due to the closure of certain factories in fish feed producing countries (e.g., Ghana, Nigeria). The severe disruption to international vessel traffic also disrupted the supply of inputs. Similarly, the strengthening of controls at the port of Cotonou has led to a sharp increase in the time taken to remove containers (FENAPIB, 2020). The primary effect is the disruption of stocks leading to a high unavailability of inputs both at the national and regional levels. Similar constraints on access to inputs have been reported in other West African countries, such as Ghana (Ragasa et al., 2021) and Nigeria (Belton et al., 2021).

An increase was also in feed transportation costs between the SBZ and the non‐SBZ. The corollary of this is the high cost of fish feed with additional costs of the transfer price, which can go up to USD 25.69 per bag of feed (FENAPIB, 2020). Overall, fish feed is imported feed with no fixed costs, depending on the manufacturer and the type of feed, with price ranging from USD 1.5 to USD 2 per kg. In addition to this, there are the additional costs associated with the search for fish feed by aqua‐farmers. Note that 59% of fry orders placed by aqua‐farmers were not delivered. Their infrastructures remained unfarmed, due to the difficulty of access to fry. The direct consequence is that their production forecast did not meet 50%, thus distorting the production targets (FENAPIB, 2020). At the national scale, the aquaculture production has indeed decreased from 5114 tonnes in 2019 to 3030 tonnes in 2020. Note that 25% of aqua‐farmers have switched from commercial feed to local feed that does not necessarily meet the nutritional needs of the fish. This is because the identification and use of locally available ingredients meeting the nutritional needs of fish remain a major challenge in the country. In the same way, approximately 31% of SSAPs have reduced the fish feeding frequency (FENAPIB, 2020). Furthermore, for successful aquaculture, we have to overcome the problem of poor‐quality seeds and high cost of quality feeds for Beninese SSAPs to have economically viable businesses. The same pattern has been observed in India, where about 27% of shrimp farmers who had prepared their ponds for stocking did not stock them because of the difficulty and uncertainty of obtaining high‐quality seed and continuous supplies of other inputs (Kumaran et al., 2021).

### 3.2 Labour markets

The AEFs are owned by both men and women, but at a much higher proportion for men (92%). Small‐scale and subsistence aquaculture activity is mainly practiced in the southern part of the country in localities such as Akpro‐Missérété, Allada, Dangbo, Lokossa, Ouidah, Sèmè‐Kpodji, and Zè. Ouémé is the territorial department where aquaculture activity is most practiced, especially in localities such as Dangbo (47%), Sèmè‐Kpodji (15%), and Akpro‐Missérété (7%) (Table 2). The advent of COVID‐19 has led to a drop in the sales turnover of SSAPs, resulting in staff reductions and increased unemployment. The COVID‐19 pandemic limited the job opportunity for 48% of youth in AEFs. Indeed, SSAPs are reported to be experiencing a shortage of manpower (38%), lack of casual workers (11%), and staff diminution (6%) because employees were having difficulty in finding transport to their work place. Employers who have been able to maintain their work pace have instituted distancing measures for on‐site technicians with staff rotation (26%). About two respondents acknowledged having established physical distance from customers. Similarly, the same percentage of SSAPs acknowledged being reluctant to hire casual workers. And only one SSAP reported a case of refusal of some technicians/employees to come and work on site. A similar situation has been reported in India, where, due to movement restrictions, many shrimp producers have experienced an inability to find skilled labour, and this has severely affected hatchery performance and led to a drop‐in seed production (Kumaran et al., 2021). So, the evidence shows that COVID‐19 has highlighted the vulnerability of many groups working in or dependent on the seafood‐related sector (Love et al., 2021).

**TABLE 2:** Profile of small‐scale and subsistence aquaculture producers (SSAPs) along with their locations in the sanitary barrier zone (SBZ) and implications of coronavirus disease 2019 (COVID‐19) on their labour market

		Territorial department					
Characteristics	Modalities	Atlantique (n = 20)	Ouémé (n = 68)	Mono (n = 10)	Total (N = 98)	Proportion (%)	Confidence interval
Gender	Men	16	64	10	90	92	5.42
	Women	4	4	0	8	8	5.42
COVID‐19's implication on the workforce	SSAP experiencing a shortage of manpower	15	22	0	37	38	9.60
	Lack of casual workers	11	0	0	11	11	6.25
	Distancing the technicians on site and staff rotation	2	23	1	26	26	3.92
	Staff diminution because employees are having difficulty in finding transport	2	0	4	6	6	4.75
	Reticence to hire casual workers	2	0	0	2	2	2.80
	Refusal of technicians/employees to come and work on site	0	1	0	1	1	1.99
	Distancing of customers	0	0	2	2	2	2.80

*Note*: The numbers n and N in brackets correspond, respectively, to the number of informants per territorial department and the sample size. Proportions and sample sizes were generated based on descriptive statistics.

### 3.3 Aquaculture production

The production facilities used by the responding SSAPs are ponds, cages and tanks (Table 3). These facilities are used by both men (≥86%) and women (≥7%). The SSAPs face enormous challenges in ensuring the profitability of their aqua‐farming facilities. The difficulties inherent to production are the high cost of fish feed (17%), rising input costs and the rising transport costs (49%), high labour costs (3%), lack of financial resources (15%), and lack of customers and collapsing sales due to the closure of restaurants and hotels (8%). The difficulties mentioned by women are mainly the high cost of fish feed, rising input and transport costs, and lack of financial resources.

**TABLE 3:** Gender‐based distribution of aquaculture facilities in small‐scale and subsistence aquaculture producer (SSAP) farms and coronavirus disease 2019 (COVID‐19)‐related difficulties affecting aquaculture production

		Men			Women			Total		
		Number	%	CI	Number	%	CI	Number	%	CI
Aquaculture production facilities	Pond	84	86	6.93	7	7	5.10	91	93	5.10
	Cage/enclosure	9	9	5.72	1	1	1.99	10	10	5.99
	Tank/raceway	15	15	7.13	4	4	3.92	19	19	7.83
Difficulties	High feed costs	14	14	6.93	3	3	3.41	17	17	7.50
	High input costs and high transport costs	44	45	9.85	4	4	3.92	48	49	9.90
	High labour cost	3	3	3.41	0	0	0.00	3	3	3.41
	Lack of financial resources	14	14	6.93	1	1	1.99	15	15	7.13
	No customers	3	3	3.41	0	0	0.00	3	3	3.41
	No reason	4	4	3.92	0	0	0.00	4	4	3.92
	Sales slump due to the closure of restaurants and hotels	8	8	5.42	0	0	0.00	8	8	5.42

*Note*: The symbol % refers to the proportion. Proportions and sample sizes were generated based on descriptive statistics performed with RStudio software. The 95% confidence intervals, however, were calculated using the Excel spreadsheet.

Abbreviation: CI, 95% confidence interval.

The difficulty of access to inputs (e.g., fish feed, seed, pond supplements) led to a disruption of fish growth and therefore, of the production cycle. In many aqua‐farms, the aquaculture production cycle is no longer followed as there is a disruption of fish growth. There is disruption due to the failure to remove fry on the correct date because fish that have reached the end of their cycle have not been released. This resulted in an increase in the cost of seed production and fish grow‐out (FENAPIB, 2020). On many farms, poor seed growth has been observed due to the unavailability of imported commercial feed for fry feeding. In Tunisia, the long‐term effects of COVID‐19 are expected to disrupt the rearing cycle of aquaculture species (FAO, 2020c). FAO (2020b), in another assessment of COVID‐19, indicated that 100% of the farms were experiencing, or expecting that its impact would have negative consequences on the whole aquaculture production.

Aqua‐farmers have also been confronted with a decrease in the demand for seed due to the fact that stakeholders avoid contact with each other. The aqua‐farmers located outside the SBZ have had difficulties in supplying seed. A 25% reduction in the turnover of fry producers was reported by most of the SSAPs. For fish grow‐out, a decrease in turnover of up to 60% has been observed in many aqua‐farms. In addition to the above, difficulties in the routing and delivery of orders already placed by aqua‐farmers were observed. Aqua‐farms that have experienced poor sales have experienced additional feed consumption and increased risk of fry loss due to lack of appropriate facilities (FENAPIB, 2020). In addition, high mortalities have been experienced due to the lack of capacity to monitor large seed (FENAPIB, 2020).

### 3.4 Processing of aquaculture products

Among our informants, very few WFWs (2%) have continued processing aquaculture products for commercial purposes during this coronavirus pandemic. Their main processing process is fish and shrimp smoking. However, the establishment of cold storage facilities and the promotion of the internal market for the processed products (Kumaran et al., 2021) can be envisaged. The situation of post‐harvest aquaculture products is evolving with a recent installation of processing ovens in the locality of Ifangni. Another fish smoking unit based on the FAO‐Thiaroye processing technique, consisting of Thiaroye processing ovens, has been set up in the village of Gbaglan‐Ganfan in the commune of Avrankou (ATDA, 2019; MAEP, 2011). Cold‐storage of post‐harvest aquaculture products is worth developing as well. Fish processing is mainly done by the women in charge of the AEFs. The main difficulty inherent in the processing of aquaculture products is the reduction in the sale of braised fish due to the closure of shops and bars, the majority of which opt for the sale of braised fish. The reduction in fish and shrimp smoking activity was also reported (FENAPIB, 2020). Therefore, the repercussion of this state of affairs has been a reduction in sales to the fishers and aqua‐farmers who supply these processors. For many of the SSAPs, a drop‐in turnover of up to 50% was observed in the aquaculture products processing sector (FENAPIB, 2020). Fortunately, the preference for storage infrastructure has been flagged as a main solution (Mangano et al., 2022), and this will need to be considered in any scheme for mitigation measures.

### 3.5 Access to local and international markets

**FIGURE 4:**
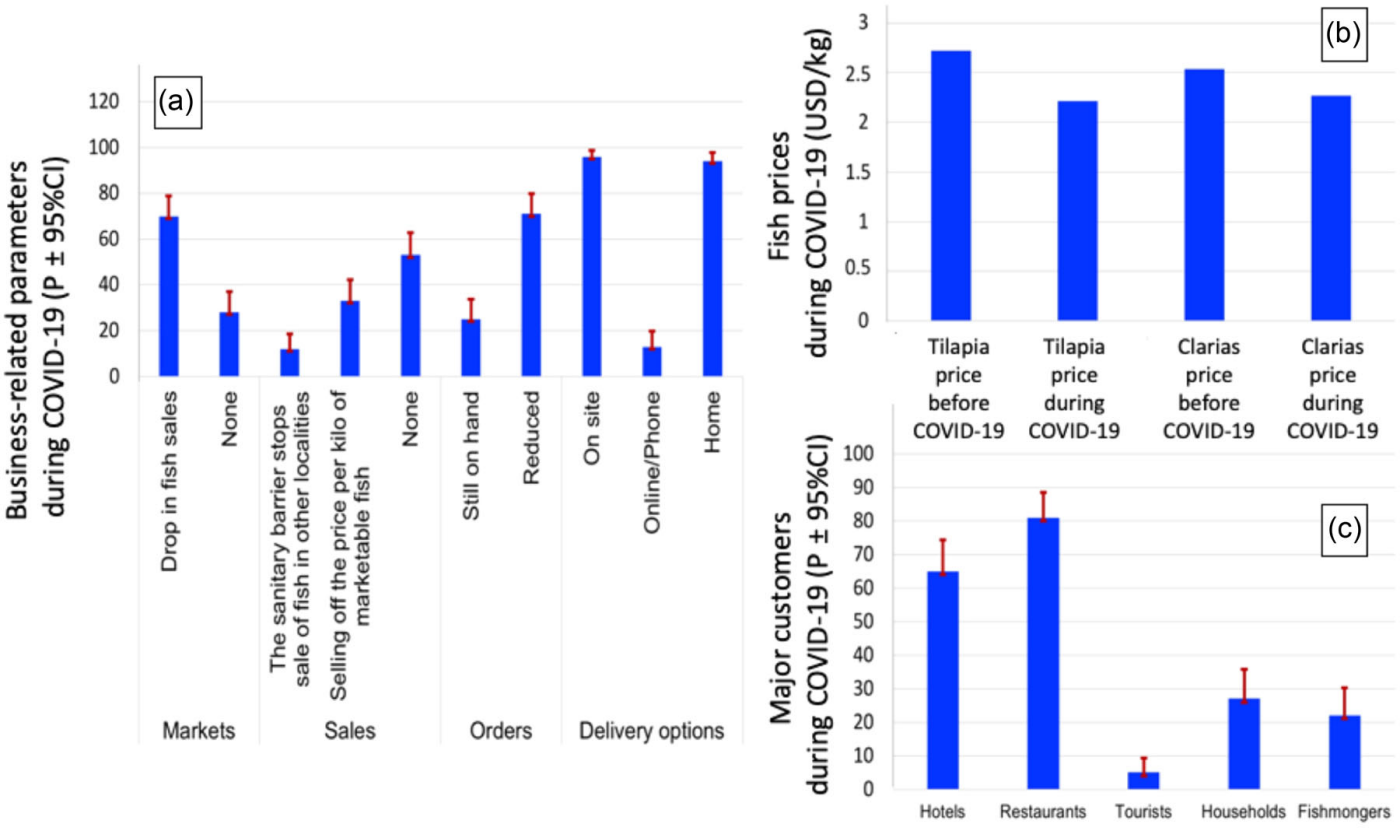
SSAPs’ business‐related parameters and percentage of major customers during COVID‐19 (a and c) along with the impacts of COVID‐19 on the commercialization of their production (b). Plots (a) and (c) are based on the proportions and the corresponding 95% confidence intervals. Plot (b) was made on the basis of the average sales prices per kilogram of fish before and during COVID‐19. Abbreviations: CI, 95% confidence interval; COVID‐19, coronavirus disease 2019; P, proportion; SSAPs, small‐scale and subsistence aquaculture producers; USD, United States dollar

Within the aqua‐farms, the most immediate impact of COVID‐19 was the drop‐in fish sales, and this was mentioned by 70% of the respondents (Figure 4a). However, 28% of the actors acknowledged that they had not felt the effect of COVID‐19 on the market for the sale of aquaculture production. The drop‐in demand for aquaculture products has been seen at the global level (FAO, 2020b). In the same way, in Malaysia, findings from an initial national survey indicate that market demand and logistical bottlenecks are major constraints for aquaculture activities (Azra et al., 2021; Waiho et al., 2020). Many SSAPs acknowledged that the SBZ established has greatly impeded the sale of fish in other towns. In addition, the SBZ put in place favoured the increase of fish transaction costs between the aqua‐farmer and the buyers/distributors. In many AEFs, there has also been a 20–30% reduction in the availability of commercial fish for sale (FENAPIB, 2020). Many SSAPs (71%) acknowledged that fish orders were reduced. These same observations were made among fishers in the Northeast United States, who nearly all reported a loss of income (largely driven by disruptions of export markets), the loss of restaurant sales, and a resulting decline in seafood prices (Smith et al., 2020). In the same way, other data suggest that consumer demand for seafood from restaurants dropped by upwards of 70% during lockdowns (White et al., 2021). However, in Benin, during COVID‐19, fish deliveries are made either on site (96%), online or by telephone (13%) or directly to the customer's home (94%).

The distribution and marketing of aquaculture products saw a 20%–30% drop in turnover in many farms (FENAPIB, 2020). There has also been a slump due to the general economic gloom created by the health crisis and restrictions on all festivities and celebrations that consume AEFs’ products. The situation has led not only to a reduction in the profit margin of aqua‐farmers but also to an increase in the selling price of fish in some localities. Indeed, there was a net decrease in the selling price per kilogram of fish. For example, 1 kg of catfish (*Clarias gariepinus* B.) went from USD 2.54 to USD 2.27. The price per kilogram of tilapia (*Oreochromis niloticus* L.) has decreased from USD 2.72 to USD 2.22 (Figure 4b). The main customers are hotels, restaurants, tourists, households, and WFWs. Customers in the tourist category have the lowest percentage (<10%), and this is an indication that COVID‐19 has greatly reduced the number of customers in this category (Figure 4c). Many other challenges related to the fish market were mentioned, including difficult market access, public reluctance, and the high cost of raw materials.

The results of LDA are summarized in Table 4. Almost all discrimination coefficients are far from zero. All the latent variables considered are therefore important for discrimination among all territorial departments and are thus considered in the model. So, it emerges that, if COVID‐19 is expected to continue, the above‐mentioned problems should be felt in all territorial departments of the country. However, the modalities that best explained the variations per department are “no sale‐related impact,” “selling off the price per kilogram of marketable fish,” and “online/phone delivery option.” Thus, in the future, no impact on the sale of production is to be expected, but there will be a drop in the price per kilogram of fish. The adoption of digital tools in the sale of products should increase. The online or phone call delivery options will take precedence over onsite deliveries. To a lesser extent, COVID‐19 will have very little impact on the market for aquaculture products. In other words, all variables being equal, the "no market‐related impact" modality will score higher than the "market‐related impact" modality by 0.682063. Thus, COVID‐19 will have a lesser impact on aquaculture byproducts. This hypothesis has been previously put forward as the aquaculture sector could enhance the resilience of the global seafood system by increasing the diversity of species harvested and production locations (Troell et al., 2014), but the hypothesis has not yet been sufficiently tested. However, the alternative seafood networks have been suggested to help contribute to the systemic resilience of the global seafood system (Stoll et al., 2021). Therefore, local, national, and international market solutions should be taken up to ensure the livelihoods and nutrition and health of both small‐scale fishers (Knight et al., 2020) and SSAPs.

**TABLE 4:** Modelling the future impacts of coronavirus disease 2019 (COVID‐19) on small‐scale and subsistence aquaculture producer (SSAPs) in the territorial departments of Benin

	Coefficients of linear discriminants	
Explanatory terms	LD1	LD2
No market‐related impact	0.682063	1.1325124
No sale‐related impact	**4.59388438**	−1.6520956
Selling off the price per kilogram of marketable fish	**4.27204401**	−1.8519498
Orders reduced	−0.53704656	−0.1670235
Orders still on hand	−0.28297441	−0.2199469
On site delivery option	−1.47146352	1.5884545
Online/phone delivery option	−1.92121104	**−2.2112889**
Home delivery option	−0.31222386	0.6358333
Export delivery option	−0.07292301	1.50795

*Note*: The LD are the coefficients of the canonical variable derived from LDA statistical inferences. Bold values represent the modalities that best explain variations per department.

### 3.6 Advice and support received by SSAPs

During the coronavirus pandemic, many SSAPs have been supported and assisted by NGOs (CAIRE BENIN, PLAN BENIN, Pain Pour Le Monde), state services (ATDA “Territorial Agency for Agricultural Development,” DDAEP “Departmental Office of Agriculture, Livestock and Fisheries,” Social Centers) and municipal services. However, 15% of the informants reported an insufficient assistance from aquaculture extension services agents, probably because they did not receive either a visit or phone call from the appropriate government and non‐government services. Advice received included protective measures against COVID‐19, how to cope with COVID‐19, and fish farm management during COVID‐19. Protective measures advised by different organizations included the use of masks or face masks, handwashing kits, social/physical distancing, changing or rotating shifts to reduce the number of people working at the same time and temperature control. Furthermore, SSAPs responded differently as to whether their activity would survive if COVID‐19 were to continue. Indeed, the survival periods mentioned by the informants were 1 month, 2 months, and 6 months for some and 1 year for others. Across all departments, the majority of SSAPs agreed that their activity would not continue if COVID‐19 is expected to last more than 6 months.

**FIGURE 5:**
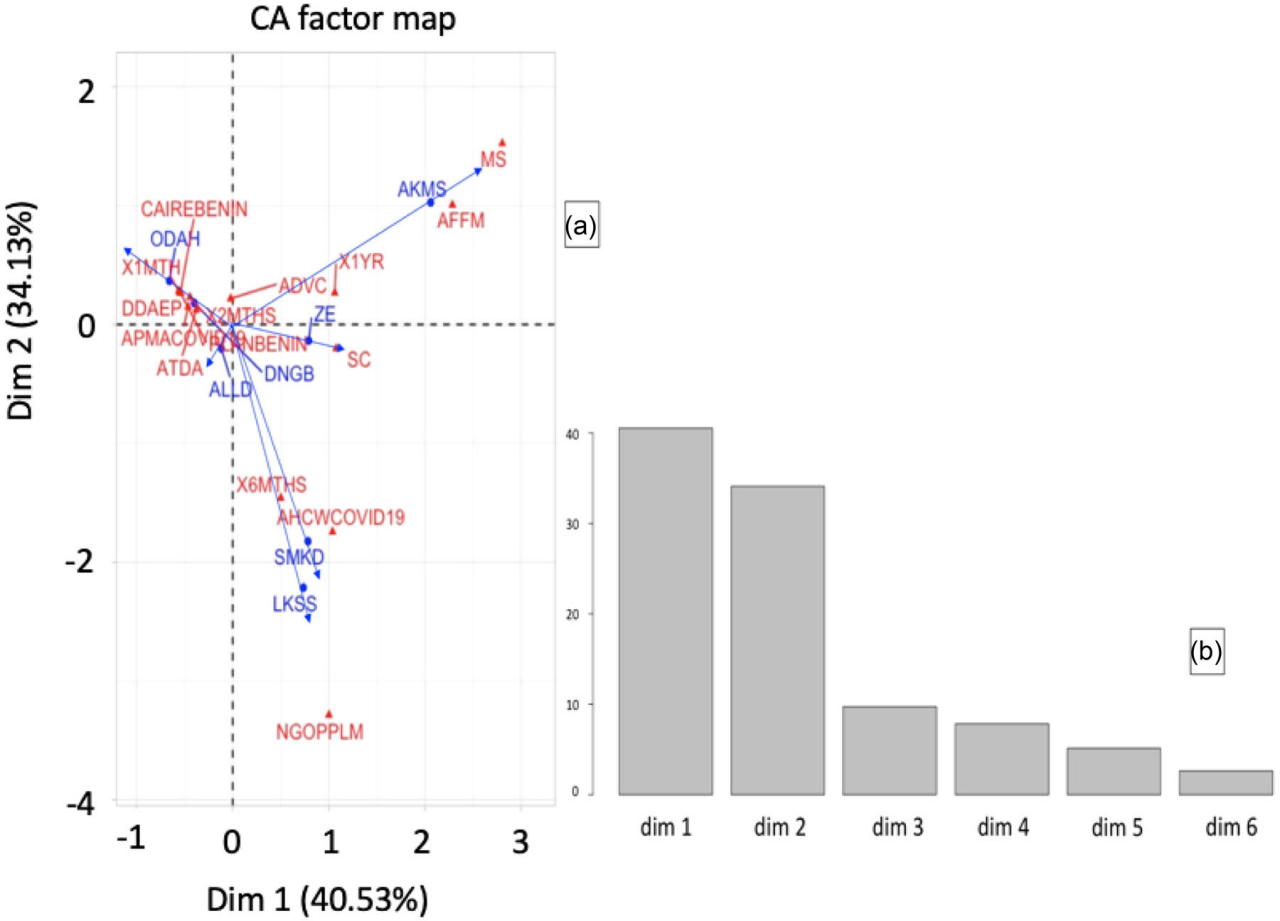
Biplot from FCA (a) showing the relationship between the territorial departments, reasons why supports/advices are needed, support/advice providers and type of support received by SSAPs during COVID‐19. The plot (b) gives the eigenvalue percentage of variance cumulative percentage of variance. The blue colour represents the localities, while the red colour refers to the other variables considered in the inference. Abbreviations: ADVC, advice; AFFM, advice on fish farm management; AHCWCOVID19, advice on how to cope with COVID‐19; ALLD, Allada; AKMS, Akpro‐Missérété; APMACOVID19, Advice on protective measures against COVID‐19; COVID‐19, coronavirus disease 2019; DNGB, Dangbo; FCA, factorial correspondence analysis; LKSS, Lokossa; ODAH, Ouidah; MS, MunicipalServices; SC, SocialCenter; SMKD, Sèmè‐Kpodji; SSAPs, small‐scale and subsistence aquaculture producers; ZE, Zè; 1MTH, 1 month; 1YR, 2MTHS, 2 months; 6MTHS, 6 months; 1YR, 1 year

The FCA showed that most of the information provided by the SSAPs on the support received was concentrated on dim 1 and dim 2, that is, 41% and 34%, respectively (Figure 5). The analysis of the FCA biplot was done on the basis of the column profile which considered the variables and the row profile which concerned the localities. For the column profile, the variables “municipal service” and “advice on fish farm management” on axis 1 and the variable “6 months” on axis 2, gave the strongest contributions (i.e., ≥10%) and the strongest cos 2 (i.e., ≥50%). For the row profile, based on the highest contributions (i.e., ≥ 15%) and the highest cos 2 (i.e., ≥ 50%), the best represented localities on Axis 1 were Akpro‐Missérété and Dangbo, while the best represented on Axis 2 were Lokossa and Sèmè‐Kpodji. So, SSAPs in localities such as Akpro‐Missérété and Dangbo acknowledged that they received advice from municipal services on fish farm management during COVID‐19. The SSAPs of Lokossa and Sèmè‐Kpodji believed that they would not be able to carry out fish production activities if COVID‐19 lasted more than 6 months. Although the responses of SSAPs from these regions appear to be different (or not) from those of others, various levels of mitigation strategies need to be adopted as suggested by a global‐scale assessment paper (Mangano et al., 2022).

### 3.7 Feasible mitigation initiatives

The advent of the coronavirus resulted in a multidimensional and multifactorial shock that has not spared the fisheries sector. In the aqua‐farms, the negative impacts were observed with regard to the supply of inputs, the production cycle, the processing of fish products, the labour market, and the marketing of products. The AEFs are therefore subject to a confluence of factors that can jeopardize their survival. Employees dependent on multifaceted livelihoods will be increasingly vulnerable. The selling price of aquaculture production has fallen in almost all AEFs as a result of reduced demand. There is an obvious need to support both large‐ and small‐scale aqua‐farms. So, if the government wants to ensure the long‐term stability of SSAPs, support should be provided by organizing, together with the stakeholders, the ordering, and transportation of inputs (fish feed, vaccines, antibiotics, hormone, etc.). Alleviating customs formalities (reducing transit time and the cost of customs fees and removal charges) on imported feed at the port of Cotonou would be of great help to SSAPs. The public authority can also negotiate transport facilities with the supplier countries concerned and, as well, give special exemptions for the removal of inputs. Lightening the sanitary barrier crossing for freight trucks is strongly recommended to facilitate transportation of SSAPs’ inputs and outputs. The public and the private sector could also give supports to SSAPs in the development of local feeds with high growth potential. There is also a need to raise awareness of the fact that the health crisis does not prevent production activities. Support in the form of supplies and equipment could be provided to stakeholders to facilitate compliance with barrier measures against COVID‐19. The authority's contribution may also extend to supporting SSAPs with subsidized fish feed. Large‐scale fish producers need to take adequate measures to increase the availability of seed to supply SSAPs. In this regard, one of the key actions planned by the authority in its plan to mitigate the effects and impact of COVID‐19 in the agricultural sector for food and nutritional security is to facilitate access to seeds (MAEP, 2020).

The advent of this pandemic requires rethinking and innovating the capacity and resilience of SSAP communities. The importance of the commitment of the private sector in this health crisis is no longer in doubt. The private sector also has a central role to play in facilitating aqua‐farmers’ access to low‐cost feed. COVID‐19 provides the opportunity for the private sector to accompany SSAPs in their search for alternative strategies for selling processed fish and shrimp. However, for the private sector, it is important that the investment should also gain economic or social benefits. The role of the partners here on how to convince the private sector of the importance and benefits of investing for the benefit of the SSAPs is needed. Large‐scale AEFs have an important role to play by making technologies available as much as possible to SSAPs. Innovation in digital technology (IDT) can help online platform, e‐commerce, and e‐payment development to connect the actors. The SSAPs have a responsibility to make forecasts, and offer their products on the marketplace via digital tools to create a market linkage. Indeed, IDT will provide a market information system platform to inform SSAPs in real time on the prices of products on the markets, link buyers and actors, bring together supply and demand, give SSAPs information on the availability of quality and affordable inputs, and establish a modern call centre to allow actors to access any expertise in case of need in their AEF. In addition, SSAPs have to digitalize their mechanisms to reach the greatest number of communities to address food security issues and awareness about the COVID‐19 pandemic. The use of social media can contribute to the good aquacultural practices and allow access to markets for SSAPs. The internet should now be a universal right to facilitate digital e‐commerce so as to leave no one behind. The use of digital tools will also help SSAPs raising the problems they are facing to policy decision makers. Public services must include digital advice to retain SSAPs. Digital platforms must alert on the technical itineraries, good post‐harvest practices, so on. In addition, SSAPs will have to subscribe to insurance and social protection schemes in order to ensure the sustainability of their activity. Public services must also favour the AEFs’ access to credit through a partnership with financial agencies. An effort must be made to reduce the illiteracy of SSAPs in order to facilitate the use of digital platforms. On the other hand, since the vulnerability of the homestead aquaculture farming and other emerging aquaculture is no longer in doubt during the current COVID‐19 pandemic, it would be necessary for SSAPs to invest in the use of indigenous technologies. Thus, alternative local technologies such as “Whedo” (Imorou‐Toko et al., 2007) and “Acadja‐enclos” (Konan‐Brou & Guiral, 1994), based on the use of artificial habitats, will help SSAPs to anticipate, mitigate, and resist possible future disturbances. Another endogenous option based on integrated agro‐aquaculture systems that upgrade plant and animal wastes while enriching the silt and the water for use as fertilizer in the fields might also help increase the resilience of SSAPs. The use of such methods might enhance resilience to multiple stressors (e.g., COVID‐19, anthropogenic‐driven threats and climate change) by providing different market options under the COVID‐19 pandemic. In addition, the adoption of endogenous phytotherapy (Chong et al., 2020) represents another alternative to prevent and cure aquaculture pathologies. Moreover, there is an urgent need to identify and valorize local feeds with high growth potential for fish.

The current COVID‐19 pandemic is an opportunity to economically empower women in the AEF sector. Women's contribution to aquaculture production deserves to be considered in various development policies because, for example in Africa, women's roles are diverse and dynamic, they vary according to regions, economy, and culture and span the entire fisheries value chain (Bradford & Katikiro, 2019). WFWs should not be marginalized in terms of resources and training (Thorpe et al., 2014). Furthermore, worldwide, the youths are not much concerned with aquaculture. The question we have to ask ourselves now is how best can their attention be drawn or how best can the attention of youth be drawn to the aquaculture sector. The SSAPs will thus have to limit job losses and highlight the effects of the pandemic on the mental health and mental resilience of employees, as no much attention has been given to the mental health issues in Africa.

## 4 CONCLUSION

The COVID‐19‐related health crisis has caused panic and major difficulties for stakeholders in the aquaculture sector. It is now clear that SSAPs are struggling to stay afloat with large price increases for inputs, considerable decreases in sale value, and a change to the way that customers are engaging. These difficulties due to the restrictive measures that were in force are perceptible at the level of all the components and functions of the sector. Imports and supply of inputs (fish feed, seed, pond supplements, etc.) are the major difficulties observed at the national level. The scarcity and high cost of feeds account for a large part (60%–70%) of fish production costs. The scarcity and high cost of feed have indeed affected the availability of fish feed, resulting in stunted growth of fry and marketable fish. The closure of places of consumption (refreshment bars, and restaurants) has led to a decrease in the processing and marketing of aquaculture products. A drop‐in revenue of between 20% and 30% was reported by all SSAPs.

The SSAPs need support in ensuring the supply of affordable inputs, through funding, greater access to credit, and regulatory import arrangements for freight, and equipment. In fact, initiatives should be taken to play a key role in putting economies back on the path of inclusive growth, since the short‐ and long‐term effects of the COVID‐19 risk are further marginalizing many SSAPs. Their role in the development of aquaculture is clear, and if efforts are not made to support them, this trend is likely to fade. The SSAPs who may have lost their business or jobs are likely to seek government assistance. Accompanying measures could therefore make it possible to overcome not only these challenges, but also to preserve aquatic natural resources. The use and adoption of both (i) modern options (e.g., based on IDTs) for organizing SSAPs into networks and (ii) traditional options (e.g., based on Whedo, Acadja‐enclosure, agro‐aquaculture, good quality local feeds, and endogenous phytotherapy) would help increase the resilience of SSAPs during this COVID‐19 pandemic and towards potential future disruptions. Investment in and wider availability of online technologies will be crucial for navigating this and future crises, with boosting national literacy of SSAPs at the core of this challenge, plus increasing interest from the youth in starting aquaculture ventures to prevent a labour drain in this sector. It is also urgent to bring to light the effect of the pandemic to AEF employees’ mental health or mental resilience and social protections after losing jobs. In addition, there is a need and an opportunity to increase women's contribution to food security through their conversion to aquaculture, rather than just post‐harvest processing of fisheries products.

## AUTHOR CONTRIBUTIONS

Toundji Olivier Amoussou: Conceptualization; Data curation; Formal analysis; Investigation; Methodology; Project administration; Resources; Software; Validation; Writing – original draft; Writing – review & editing; Comlan Eugène Dessouassi: Investigation, Resources, Writing – review & editing; Dorothé Ngondjeb Yong: Data curation, Methodology, Validation, Writing – review & editing; Siméon Mahougnon Fagnon: Investigation, Writing – review & editing; Luc Houngbe: Investigation, Resources, Writing – review & editing; Vinsoun Millogo: Writing – review & editing; Ibrahim Imorou Toko: Data curation, Writing – review & editing; Emmanuel A. Frimpong: Conceptualization, Data curation, Formal analysis, Methodology, Supervision, Validation, Writing – review & editing.

## CONFLICT OF INTEREST

The authors declare no conflict of interest.

### ETHICS STATEMENT

This investigation was conducted in accordance with fundamental ethical principles and standards, international conventions and declarations on ethical considerations and intellectual property rights. The participants were informed about the study and their possibilities to resign. Respect for anonymity and confidentiality was considered during the investigation.

### PEER REVIEW

The peer review history for this article is available at: https://publons.com/publon/10.1002/aff2.78.

## Supporting information


**TABLE S1** Survey form: COVID‐19 and Aquaculture in Benin

## Data Availability

The data that support the findings of this study are available on request from the corresponding author T.O.A. The data are not publicly available due to privacy/ethical restrictions.
