# Supplementary material for: The multifactorial aquaculture‐related COVID‐19 shock in Benin, West Africa: A socio‐economic perspective of mitigating the disruptive impacts on the small‐scale and subsistence producers
Source: Aquac Fish Fish. 2022 Oct 24;2(6):507–21. doi: 10.1002/aff2.78 (PMC9874826; doi:10.1002/aff2.78)
Supplement: Supplementary file 1 — TABLE S1 Survey form: COVID‐19 and Aquaculture in Benin [file AFF2-2-507-s001.docx]

**Supplementary material**

**TABLE S1** Survey form: COVID-19 and Aquaculture in Benin

| 1 | Contacts of the aqua-farm’s Resource Person(s): |  | | | | | |
| --- | --- | --- | --- | --- | --- | --- | --- |
| 2 | Sector of activity (Tick the answer(s)): |  | Aquaculture |  | Fishing |  | Processing |
| 3 | Type of activity (Tick the answer(s)): |  | Ponds |  | Inland fishing |  | Freezing /Deep-freezing |
|  | |  | Cages/Enclosures |  | Industrial sea fishing |  | Smoking |
|  |  |  | Tanks/Raceways |  | Artisanal sea fishing |  | Salting |
|  |  |  | Aquarium fish |  | Restocking fish |  | Fish fillets |
|  |  |  | Other (Please specify:) |  | Other (Please specify:) |  | Other (Please specify:) |
| 4 | What fisheries products are sold? Tick the answer(s) | | | | | | |
|  | |  | Fish | | | | |
|  |  |  | Shrimp | | | | |
|  |  |  | Shellfish | | | | |
|  |  |  | Caviar | | | | |
|  |  |  | Other (Please specify:) | | | | |
| 5 | Has COVID-19 affected your input supplies (Electricity, Fuel, Fresh Fish, Bait, Ice, Feed, Fry, Vaccines, Packaging, etc.)? Yes or No. If yes, specify the type of impact: | | | | | | |
|  | | | | | | | |
| 6 | What were the prices of your inputs (Electricity, Fuel, Fresh Fish, Bait, Ice, Feed, Fry, Vaccines, Packaging, etc.) before and during COVID-19? Please specify: | | | | | | |
| Input 1 (please specify) : | | Before COVID-19 | |  | During COVID-19 |  | Other inputs (Please specify:) |
| Input 2 (please specify) : | | Before COVID-19 | |  | During COVID-19 |  |  |
| Input 3 (please specify) : | | Before COVID-19 | |  | During COVID-19 |  |  |
| Input 4 (please specify) : | | Before COVID-19 | |  | During COVID-19 |  |  |
| Input 5 (please specify) : | | Before COVID-19 | |  | During COVID-19 |  |  |
| 7 | What are the main market and sales impacts? Please specify the type of impact: | | | | | | |
|  | | | | | | | |
| 8 | Who are your main customers? Tick the answer(s) | | | | | | |
|  | |  | Hotels | | | | |
|  |  |  | Restaurant | | | | |
|  |  |  | Tourists | | | | |
|  |  |  | Other (Please specify:) | | | | |
| 9 | Are the orders always on time? Yes or No, please specify: | | | | | | |
|  | | | | | | | |
| 10 | How are deliveries made to your customers? Tick the answer(s) | | | | | | |
|  | |  | On-site delivery | | | | |
|  |  |  | On-site delivery after an online order | | | | |
|  |  |  | Home delivery | | | | |
|  |  |  | Export (commercial flights) | | | | |
|  |  |  | Other (Please specify:) | | | | |
| 11 | Specify difficulties inherent in the financial management of the farm/company/activity that are due to COVID-19: | | | | | | |
|  | | | | | | | |
| 12 | Specify the implications for the operational management of the farm/business/activity (Sorting, Handling, Sales, Transport, Alternation of human resource (staff), Distancing of technicians/employees, Frequency of feed distribution, frequency of sea trips, etc.): | | | | | | |
|  | | | | | | | |
| 13 | Do you think that COVID-19 could seriously impact the aquaculture/fisheries market if it were to persist on: | | | | | | |
|  | 1 month (Please tick and specify): | | | | | | |
|  | 2 months (Please tick and specify): | | | | | | |
|  | 6 months (Please tick and specify): | | | | | | |
|  | 1 year (Please tick and specify): | | | | | | |
| 14 | Specify other (aquaculture/fish) diseases faced by the farm/business: | | | | | | |
|  | | | | | | | |
| 15 | Are you facing human resource (labour) shortages due to COVID-19? Yes or No, If Yes Please specify: | | | | | | |
|  | | | | | | | |
| 16 | Did you receive advice/support from the aid/extension services? Yes or No, If Yes, Please | | | | | | |
|  | | | | | | | |
